# Clinical Characteristics and Prognosis of Concomitant Primary Biliary Cholangitis and Autoimmune Diseases: A Retrospective Study

**DOI:** 10.1155/2021/5557814

**Published:** 2021-03-17

**Authors:** Yuwei Liu, Kai Han, Chen Liu, Fangfang Duan, Jun Cheng, Song Yang

**Affiliations:** ^1^Emergency Department, Beijing Ditan Hospital, Capital Medical University, Beijing 100015, China; ^2^Institute of Infectious Diseases, Beijing Ditan Hospital, Capital Medical University, Beijing 100015, China; ^3^Center of Hepatology, Beijing Ditan Hospital, Capital Medical University, Beijing 100015, China

## Abstract

**Objectives:**

Diagnosis and treatment of primary biliary cholangitis (PBC) are often complicated by hepatic and/or extrahepatic manifestations, which in turn affect the natural course and prognosis of PBC. This study evaluated the clinical characteristics and prognosis of PBC co-occurring with intrahepatic and extrahepatic autoimmune disease (AID).

**Methods:**

Clinical data of patients with PBC who were admitted to the Beijing Ditan Hospital from September 2008 to December 2014 were retrospectively reviewed, assessed for other autoimmune diseases, and analyzed statistically. All patients received ursodeoxycholic acid (UDCA) treatment.

**Results:**

Data from 505 patients were evaluated. Approximately 35.0% of patients had at least one additional AID. AIDs included Sjögren's syndrome (SS; 26.3%), autoimmune hepatitis (AIH; 7.1%), rheumatoid arthritis (RA; 1.4%), hypothyroidism (0.8%), Graves's thyroiditis (0.6%), systemic lupus erythematosus (SLE; 0.4%), and Hashimoto's thyroiditis (0.2%). No differences in response rates of UDCA were found between the PBC group and the PBC-SS group or PBC complicated with AID group (both *P* > 0.05). White blood cell (WBC, RR = 1.072, 95% CI: 1.016–1.130, *P*=0.011), platelet counts (PLT, RR = 0.995, 95% CI: 0.992–0.998, *P*=0.003), and prothrombin time and international normalized ratio (PT/INR, RR = 1.799, 95% CI: 1.010–3.206, *P*=0.046) were independent prognostic factors in patients with PBC. The overall survival time of patients in PBC-AIH and PBC-SS groups was shorter than that of those with PBC (*P* < 0.001).

**Conclusions:**

AIH was the most common in hepatic comorbidity. SS was the most frequent extrahepatic comorbidity. WBC, PLT, and PT/INR were independent prognostic factors in patients with PBC. AID coexisted with PBC impaired patients' survival.

## 1. Introduction

Primary biliary cholangitis (PBC) is a granulomatous autoimmune disease (AID) characterized by portal tracts inflammation due to lymphocyte infiltration, progressive loss of bile ducts resulting in chronic cholestasis and subsequent fibrosis, cirrhosis [1], and eventually liver failure [2]. The most common manifestations of PBC are fatigue and pruritus. The prevalence of PBC has been shown to vary globally and regionally, with prevalence rates ranging from 19 to 402 cases per million individuals [3]. PBC is an immune-mediated epithelitis with complex pathogenetic mechanisms and often concomitantly occurs with autoimmune hepatitis (AIH), systemic lupus erythematosus (SLE), undifferentiated and mixed connective tissue diseases, chronic thyroiditis, rheumatoid arthritis (RA), systemic sclerosis (SSc), and other extrahepatic autoimmune (EHA) conditions such as Sjögren's syndrome (SS) [4]. These coexisting conditions frequently increase the difficulty in making a diagnosis and treating the disease. Further, the natural history and clinical characteristics of the disease differed significantly among individual patients, with symptoms ranging from asymptomatic and slowly progressive to symptomatic and rapidly progressive [5]. During the last few decades, typical clinical presentation has changed remarkably, further increasing the complexity of the disease spectrum.

There is a significant variability in the reported incidence rates and types of PBC co-occurring with other autoimmune diseases across different countries and regions. In a study by Gershwin et al., approximately 32.0% of patients with PBC had at least one additional AID [6]. While in China Wang et al. showed that PBC overlapped with connective tissue diseases in about 46.6% of patients [7], other studies have shown that these probabilities may be underestimated [8].

Of note, the role of autoantibodies in determining patients' prognosis in PBC with SSc has been described earlier [9]. However, data related to prognosis of PBC associated with other AIDs is scarce. The study by Rigamonti et al. [9] evaluated clinical features and prognosis of PBC associated with SSc and demonstrated that liver disease has a slower progression in PBC-SSc compared with matched patients having PBC. On the contrary, a study by Floreani et al. [10] demonstrated the associations between PBC and extrahepatic autoimmune (EHA) conditions and further showed that there was no significant difference in the mean survival of patients with and without EHA conditions after the diagnosis of PBC.

Therefore, in view of the above and the paucity of information on prognosis of PBC concurrent autoimmune entities, we aimed to evaluate the prevalence, clinical characteristics, and prognosis of PBC complicated with intrahepatic and extrahepatic AIDs in China, estimate the rate of its co-occurrence, and further assess the impact of these comorbidities on its natural history on patients' survival. For this study, an established database with medical records of patients with PBC who were admitted in our hospital during the 7-year timeframe between September 2008 and December 2014 served as the primary data resource.

## 2. Methods

### 2.1. Study Design

All hospitalized patients with confirmed diagnosis of PBC who were admitted to the Beijing Ditan Hospital from September 2008 to December 2014 were retrospectively analyzed in this study. All data from patients were obtained from our collective database.

PBC was diagnosed based on the American Association for the Study of Liver Diseases (AASLD) 2009 Practice Guidelines [11]. The diagnosis of PBC was established if patients showed at least two of the following criteria: (1) presence of serum antimitochondrial antibody (AMA) titer greater than 1 : 40 (“AMA-positive”), (2) presence of abnormal alkaline phosphatase (ALP) levels (at least 1.5 times the normal value), and (3) presence of PBC-compatible histopathology. The AMA-negative variant was diagnosed in cases with abnormal alkaline phosphatase levels (at least 1.5 times the normal value), an antinuclear antibody (ANA) positivity of at least 1 : 40, and a liver histology compatible with PBC. In AMA-negative patients, biliary tree patency was determined by using ultrasound and magnetic resonance cholangiography (MRC) [12].

Patients who met the diagnosis and treatment practice guidelines for PBC as formulated by the AASLD in 2009 and patients for whom complete clinical, serological, and radiological data were available were included for participation in this study. Patients who were lost to follow-up were excluded from the analysis.

All patients were screened for the presence of AIDs, including autoimmune hepatitis (AIH) and EHA conditions, and systemic involvement was assessed. The diagnosis of EHA was confirmed when patients had at least one of the following: Sjögren's syndrome (SS), rheumatoid arthritis (RA), systemic lupus erythematosus (SLE), autoimmune thyroid dysfunction, systemic sclerosis (SSc), and polymyositis or dermatomyositis (DM). The diagnostic criteria for SS was based on the LeRoy classification [13]. The diagnosis of RA was made on the basis of revised classification criteria of the American College of Rheumatology [14]. The Bohan and Peter diagnostic criteria were followed for definitive diagnoses of dermatomyositis (DM) and polymyositis (PM) [15]. SLE was diagnosed according to the revised ACR SLE classification criteria of the Systemic Lupus Collaborating Clinics (SLICC; 2009) [16]. Revised original scoring system of the International Autoimmune Hepatitis Group (IAIHG; 1998) was used for the diagnosis of AIH/PBC overlap syndrome [17]. AIH/PBC overlap syndrome was confirmed if patients had features of both AIH and PBC. This study was approved by the Institutional Review Board of Beijing Ditan Hospital (No. JDLK 2017-044-01). The need to obtain informed consent was waived.

### 2.2. Laboratory Testing and Clinical Examinations

General laboratory tests included WBC count, neutrophil percentage (NE%), serum hemoglobin (Hb), platelet count (PLT), coagulation indicators including prothrombin time (PT), activated partial thromboplastin time (APTT), biochemical markers of liver injury including, serum alanine aminotransferase (ALT), serum aspartate aminotransferase (AST), serum alkaline phosphatase (ALP), serum gamma-glutamyl transferase (GGT), total bilirubin (TBIL), direct bilirubin (DBIL), serum albumin (ALB), total cholesterol (TC), triglycerides (TG), high-density lipoprotein cholesterol (HDL-C), low-density lipoprotein cholesterol (LDL-C), serum Ca^2+^, and serum phosphorus (PHOS).

Immunological assays for serum immunoglobulins (IgM and IgG), antinuclear antibody (ANA), anticentromere antibodies (ACA), anti-SSA/Ro-52kD, AMA, and AMA-M2 (M2 mitochondrial subfraction) were carried out. The liver function indicators were measured by using an automatic biochemical analyzer (H7600; Hitachi, Tokyo, Japan). The enzymatic or the substrate enzyme method was employed for the quantitative determination of DBIL and TBIL. Serum albumin levels were detected by using the bromocresol green (BCG) method. Serum alpha 1-fetoprotein (AFP) concentration was determined by using electrochemical luminescence immunoassay kit (E170, Roche Diagnostics, Basel, Switzerland). The reference range for serum AFP was 0.9–8.8 ng/mL.

Coagulation testing was performed by using ACL TOP 700 CTS automated coagulation analyzer (Beckman Coulter, Fullerton, California, USA) and ACL TOP HemosIL reagents and HemosIL Calibration plasma were used. PT was determined by the coagulation method. Prothrombin time and international normalized ratio (PT/INR) was calculated as (PT value of mixed plasma/PT value of calibrated plasma) ×1.42.

Abdominal ultrasonographic examinations were performed by using the Siemens color Doppler ultrasound system (Siemens, Erlangen, Germany) at a frequency bandwidth of 3.5–5.0 MHz. Prior to scanning, all patients were advised to fast for 8–12 hours. All patients underwent abdominal ultrasound in the supine and right lateral decubitus positions at full inspiration without intravenous contrast. The appearance and parenchymal echo of liver and spleen, bilateral signs of gallbladder wall, acoustic shadowing and acoustic enhancement in gallbladder, and calculi were recorded. The maximum oblique and anteroposterior diameters of the right and the left lobes were measured. Furthermore, the splenic dimensions (length, width, and thickness), thickness of the gallbladder wall, and the depth of ascitic fluid pockets were evaluated.

### 2.3. Evaluation of Liver Function

The Child–Pugh score was used to assess the prognosis of cirrhosis in patients with PBC. The score includes five clinical biochemical indices: hepatic encephalopathy (1, none; 2, grades 1–2; 3, grades 3–4), ascites (1, none; 2, mild; 3, moderate to severe), serum bilirubin (1, 17–68 *μ*mol/L; 2, 68–170 *μ*mol/L, 3, >170 *μ*mol/L), albumin (1, >35 g/L; 2, 28–35 g/L; 3, <28 g/L), and prothrombin time (1, 1–3 s; 2, 4–6 s, 3, >6 s). The total lowest and highest possible scores are 5 and 15 points, respectively. The fibrosis-4 (FIB-4) index [18] was used to assess liver fibrosis. The FIB-4 index was calculated using the following formula:

(1) $$\begin{array}{c} \text{age}\times \text{AST}\left(U/L\right)/\text{PLT}\left(\times 109/L\right)\times \sqrt{\text{ALT}\left(U/L\right)}. \end{array}$$

### 2.4. Treatment

All patients were treated with ursodeoxycholic acid (UDCA) (15 mg/kg per day) from the beginning of the study. The biochemical response after treatment was defined by a reduction in alkaline phosphatase (ALP) greater than 40% of pretreatment or up to normal levels [19] and the GLOBE score [20].

### 2.5. Data Collection and Follow-Up

The clinical data and quality indicators related to laboratory testing were obtained through the inpatient medical record system. Survival time was determined by follow-up. All patients were followed up by telephone or inpatient follow-up review until the time of death or until November 30, 2017, whichever occurred first.

### 2.6. Statistical Analyses

Data were analyzed by using the SPSS statistical software (Version 19.0. IBM Corp., Armonk, NY, USA). Continuous variables were expressed as mean ± standard deviation (SD) or median (range) and categorical variables were presented as frequencies or ratios. Nonparametric variables were presented as median with interquartile range (IQR). Comparison of outcomes between groups was performed by using Student's *t*-test or Mann–Whitney *U* test for continuous data; statistical significance for categorical data was determined by using the *χ*^2^ test or Wilcoxon rank-sum test. Survival analysis was conducted using the Kaplan–Meier curve and Log Rank test. Univariate and multivariate Cox proportional hazards regression models were performed to identify the independent risk factors influencing the overall survival. All statistical tests were two-sided, and *P* < 0.05 was considered statistically significant.

## 3. Results

### 3.1. Patients' Characteristics

A total of 505 consecutive patients with PBC (*n* = 505) were enrolled from Beijing Ditan Hospital between September 2008 and December 2014. At diagnosis, the median age of patients with PBC was 62.68 ± 12.41. Clinical comorbidities in patients with PBC are listed in Table 1. Among these, approximately 35.0% of patients with PBC (*n* = 177) had at least one additional AID, while 33.5% of them (*n* = 169) had only one AID. The AIDs included SS (26.3%), AIH (7.1%), RA (1.4%), hypothyroidism (0.8%), Graves's thyroiditis (0.6%), systemic lupus erythematosus (SLE; 0.4%), and Hashimoto's thyroiditis (0.2%). SS was associated with AIH (*n* = 3), SLE (*n* = 1), RA (*n* = 1), and hypothyroidism (*n* = 1). While in one patient AIH co-occurred with Hashimoto thyroiditis, it was associated with both SS and RA in the other.

Patients were categorized into PBC (*n* = 328), PBC-SS (*n* = 133), and PBC-AIH (*n* = 36) groups. No significant differences in age were observed between patients having PBC and AIDs and those with PBC alone. Notably, the proportion of female patients was found to be significantly higher in the PBC-AIH (121 (90.98%)) and the PBC-SS (29 (80.56%)) groups, thus indicating that female gender was significantly associated with positivity for EHA conditions (*P* < 0.05).

The absolute WBC counts (4.63 × 10^9^/L versus 3.22 × 10^9^/L, *P*=0.013), the levels of HDL-cholesterol (1.08 mmol/L versus 1.02 mmol/L, *P*=0.015), serum ALB (34.80 g/L versus 33.30 g/L, *P*=0.008), serum Ca^2+^ (2.18 mmol/L versus 2.15 mmol/L, *P*=0.004), and serum Hb (114.60 g/L versus 112.90 g/L, *P*=0.003) were found to be significantly lower in patients of the PBC-SS group compared with that of those with PBC alone (*P* < 0.05). Further, a significantly higher proportion of patients in the PBC-SS group were found to have high titers of AMA (69.9% versus 82.3%, *P*=0.007), anti-SSA/Ro-52kD (33.1% versus 48.3%, *P*=0.004), and AMA-M2 (60.8% versus 78.3%, *P* < 0.001) antibodies compared with those having only PBC (Table 2). Similarly, patients in the PBC-AIH group showed longer PT (12.30 s versus 12.80 s, *P*=0.021), elevated IgG levels (15.40 g/L versus 16.45 g/L, *P*=0.046), and high titers of ANA (58.6% versus 78.8%, *P*=0.025) compared with those having PBC (Table 3).

### 3.2. Response to UDCA

After 1 year of UDCA treatment, of the 289 patients with PBC only, 201 (69.5%) responded to UDCA. Of the 118 patients with PBC-SS, 77 (65.2%) responded to treatment. Of the 149 patients complicated with AID, 93 (62.4%) responded to treatment. No difference in response rates was found between the PBC group and the PBC-SS group (69.5% versus 65.2%, *P* > 0.05). There was no difference in response rates between the PBC group and PBC complicated with AID group (69.5% versus 62.4%, *P* > 0.05). There was no difference in the GLOBE score between the PBC and PBC-SS groups (1.83 ± 1.49 versus 1.96 ± 1.52, *P* > 0.05). Furthermore, there was no difference in the GLOBE score between the PBC group and the PBC complicated with AID group (1.83 ± 1.49 versus 1.95 ± 1.47, *P* > 0.05).

### 3.3. Univariate and Multivariate Cox Regression Analyses of Prognostic Factors in Patients with PBC

Univariate analysis of prognostic factors by Cox proportional hazard model in patients with PBC indicated that age (RR:1.028, 95% CI: 1.012–1.044, *P* < 0.001); Child–Pugh score (RR: 1.353, 95% CI: 1.251–1.464, *P* < 0.001); serum levels of TBIL (RR: 1.004, 95% CI: 1.003–1.005, *P* < 0.001), DBIL (RR: 1.005, 95% CI:1.004–1.007, *P* < 0.001), ALB (RR: 0.912, 95% CI:0.885–0.939, *P* < 0.001), Hb (RR: 0.986, 95% CI: 0.979–0.994, *P* < 0.001), and plasma creatinine (RR: 1.011, 95% CI: 1.007–1.014, *P* < 0.001); and WBC count (RR: 1.090, 95% CI: 1.034–1.149, *P* < 0.001), PLT count (RR: 0.994, 95% CI: 0.992–0.997, *P* < 0.001), PT/INR (RR: 3.706, 95% CI: 2.639–5.204, *P* < 0.001), hemorrhage from esophageal or gastric varices (RR: 1.960, 95% CI: 1.118–3.435, *P*=0.019), and hepatic encephalopathy (RR: 2.569, 95% CI: 1.680–3.927, *P* < 0.001) were found to predict the survival in patients with PBC (Table 4). Multivariate regression analysis revealed that WBC count (RR = 1.072, 95% CI = 1.016–1.130, *P*=0.011) and PLT count (RR = 0.995, 95% CI = 0.992–0.998, *P*=0.003) were independent predictors of poor prognosis in patients with PBC. Further, PT/INR (RR: 3.706, 95% CI: 2.639–5.204, *P* < 0.001) was found to strongly predict patients' prognosis in PBC (Table 5).

### 3.4. Survival Analysis

**Figure 1:**
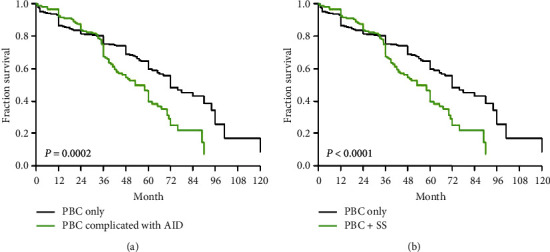
Kaplan–Meier curves for overall survival of patients with primary biliary cholangitis (PBC; black curve, median: 72 months) and those with PBC complicated with autoimmune diseases; green curve, median: 58 months. (a). Kaplan–Meier curves for overall survival of patients with primary biliary cholangitis (PBC; black curve, median: 72 months and those with PBC complicated with Sjögren's syndrome; green curve, median: 50 months) (b). AID: autoimmune disease; SS: Sjögren's syndrome.

Survival data for all patients were collected up to November 30, 2017. In the PBC group, 116 patients died, 167 of them survived, and 45 patients who were identified as lost to follow-up were traced later. One-year, 2-year, and 3-year survival rates among patients with PBC were 90%, 80%, and 75%, respectively, and the median survival time was 72 months. On the contrary, in the PBC-AID group, 91 cases died, 74 cases survived, and 12 cases were lost to follow-up. The 1-year, 2-year, and 3-year survival rates among these patients were 93%, 81%, and 66%, respectively, and the median survival time was 58 months. In the PBC-SS group, 77 patients died, 47 survived, and 9 were lost to follow-up. The median survival time was 50 months, and the survival rates for 1, 2, and 3 years were 97%, 84%, and 67%, respectively, among the patients of this group. Kaplan–Meier curves demonstrated that the overall survival time of patients in the PBC complicated with AID group and PBC-SS group was significantly shorter than that of those with PBC (*P* < 0.001; Figure 1).

## 4. Discussion

This study focused on the associations of PBC with other autoimmune entities, particularly SS and AIH. In this study, we evaluated the clinical characteristics of PBC co-occurring with intrahepatic and extrahepatic autoimmune conditions, estimated the rate of its co-occurrence with other autoimmune entities, and further assessed the impact of these comorbidities on its natural history. Most importantly, we demonstrated that PT/INR, WBC and PLT counts independently predicted prognosis in patients with PBC. Patients with PBC-AIH/PBC-SS had significantly worse prognosis; mean survival of patients in PBC-AIH and PBC-SS groups was found to be shorter than that of those with PBC.

Similar to a previous study, we observed that PBC coexisted with other AIDs in approximately 35.0% of patients [6]. However, we noted that other studies reported higher prevalence rates but were based on a smaller number of select cases [10]. This difference might probably be due to the effect of lead time bias (screening for PBC in patients with other autoimmune conditions, which could have led to an early diagnosis of asymptomatic PBC). Considering the fact that our hospital is one of the largest clinical and research centers, exclusively for liver and infectious diseases in China, the patient cohort can be representative of the entire population. Therefore, the findings of this study more closely resemble the real-world situation of patients with PBC harboring other intra/extrahepatic autoimmune entities.

In accordance with previous reports, we found that SS (26.3%) was the most commonly co-occurring extrahepatic AID. However, we noted that the reported incidence rates of SS in patients with PBC varied significantly, ranging from 33.3 to 72% [6, 7, 10, 21]. Varying incidence rates may be attributable to the different diagnostic criteria applied in different populations. While there have been 11 revised diagnostic criteria published for SS since 1965 (Copenhagen Criteria), the criteria adopted by different investigations carried out in different years were different. Further, the possibility of patients' selection bias, such as the higher incidence of SS reported by rheumatic immunology department and the preference of patients to visit rheumatic immunology department, in addition to the involvement of genetic factors linked to racial and ethnic differences, cannot be ruled out.

In this study, all patients received the UDCA treatment. We used Barcelona criteria and the GLOBE score for evaluating response to UDCA treatment and found that there were no differences in response rates between the PBC group and the PBC complicated with AID group or the PBC-SS group; these findings are consistent with the results of the study by Ni et al. in Chinese patients [3]. Owing to recent improvements in diagnosis and prognosis of PBC, the usage of UDCA in the treatment of PBC has dramatically modified its natural history [5]. A consistent proportion of PBC patients present with pruritus and/or fatigue of unexplained origin. A study showed that fatigue and pruritus might be related to worse prognosis of PBC patients [22]. However, we did not find the correlation between pruritus/fatigue and prognosis in our patients. With the approval of obeticholic acid (OCA), UDCA therapy is no longer the only option, and more and more drugs are entering clinical trials, bringing new light to patients with PBC. The recommendations of the European Society of Hepatology guidelines have given possible directions in how to stratify patients [23]. It is believed that more targeted drugs with fewer side effects will usher in a new phase of PBC patient management. However, OCA is not approved in mainland China till now.

In line with a previous study by Floreani et al. [10], we observed that female gender was significantly associated with positivity for EHA conditions in PBC. We noted a significantly higher proportion of female patients in the PBC-SS group compared with the PBC group (*P*=0.004). Reportedly, there is a striking female predominance in both PBC and PBC-SS groups. Most women were diagnosed with PBC at perimenopausal age, suggesting that estrogen deficiency may enhance their susceptibility to autoimmune disorders [24]. Moreover, estrogen is considered to be an immunoregulatory hormone, which actively regulates immune response and tissue apoptosis through its interactions with the receptors on the immune cells which ultimately affects the functioning of immune cells [25]. However, further studies are needed to understand the involvement of this hormone in the pathogenesis of AID.

In contrast to a previous study, there were no differences in IgG levels of patients with PBC and SS, despite a significant decline in their WBC counts (*P*=0.013). This could possibly be due to the small number of cases who were mostly hospitalized patients with liver disease symptoms. Currently, there is not enough evidence about the changes that may occur in lipid levels in AIDs. Nevertheless, in this study, we demonstrated a significant decline in HDL-C levels (*P*=0.015) of patients in the PBC-SS group compared with that of those in the PBC group. Corroborating this finding, a study by Zhang et al. [26] demonstrated lower serum levels of TC, HDL-C, and LDL-C in SS patients and further hypothesized that the abnormality of cholesterol metabolism in SS patients correlated with autoantibody production and disease activity [26]. Similarly, another study by Lodde et al. [27] reported lower levels of HDL-C and TC in patients with SS compared with that of those in the control group with xerostomia. Further, the prevalence of hypertriglyceridemia in patients with SS was shown to be 1.5 times higher than that of healthy controls [28]. Therefore, these findings collectively suggest the occurrence of intricate changes in the modulation of cholesterol and lipoprotein metabolism in PBC associated with SS, which in turn contributes to the disease pathogenicity. However, further studies exploring the mechanism underlying abnormal cholesterol metabolism and its impact on the immune system in PBC associated with SS are warranted. Consistent with an earlier study [29], Hb levels in patients of the PBC-SS group were found to be significantly lower than that of those with PBC (*P*=0.003). Notably, this finding highlights one of the unique clinical characteristics of PBC concurrent SS. In addition, a significant decline in albumin levels (*P*=0.008) of patients in the PBC-SS group suggests the presence of liver injury induced by PBC.

From an immunological standpoint, the presence of serum AMA and anti-M2 antibodies is pathognomonic for PBC, with SS-A/SS-B (SS-related antigen-A/B) being identified as specific for SS. In addition, one study showed that anti-SS-A⁄Ro-52kD and ACA antibodies have high specificity for PBC and can be of diagnostic relevance in antimitochondrial antibody-negative cases [30]. Expectedly, high titers of AMA (*P*=0.007), anti-SSA/Ro-52kD (*P*=0.004), and AMA-M2 (*P* < 0.001) were detected in both PBC-SS and PBC groups, with a higher prevalence in the PBC-SS group compared with the PBC group. Both SS and PBC are associated with immune-mediated destruction of the epithelia [31], and substantial evidence supports that the pathogenic mechanisms underlying the diseases are similar. Despite the presence of disease-specific autoantibodies, namely, AMA and AMA-M2 in both these conditions, direct pathogenic role has not been elucidated yet. Nonetheless, our findings indicate that the concomitant occurrence of SS and PBC may enhance immune response, thus leading to a higher antibody positivity. Therefore, earlier diagnosis of SS may provide us the possibility for tailoring better therapies to these patients.

Consistent with previous literature, a small proportion of PBC patients (7.1%) were diagnosed with PBC/AIH overlap syndrome in this study. Varying incidence rates of PBC-AIH overlap syndrome have been reported since 1988, ranging from 2% to 20% [32, 33]. This could be mainly attributed to the challenges of diagnosing two different autoimmune liver diseases in the same patient and the lack of standard diagnostic criteria for PBC-AIH overlap syndrome. Furthermore, high titers of AMA and AMA-M2 were detected in these patients, although not statistically different from those of the PBC group. However, significantly elevated levels of ANA (*P*=0.025) and IgG (*P*=0.046) were observed in these patients compared with those in the PBC group. These findings strongly suggest that a distinct group of patients with unique serologic features specific to PBC-AIH overlap syndrome do exist, a concept strongly supported by earlier studies [34, 35]. Consistent with a previous study [36], we have shown that AIH-PBC overlap syndrome might have worse clinical outcomes compared to patients with PBC alone. Further, we noted a significantly prolonged PT (*P*=0.021), indicating severely impaired hepatic synthetic dysfunction in these patients. Therefore, our findings confirm that patients with PBC-AIH progress rapidly to cirrhosis and, subsequently, liver failure [37].

Consistent with a retrospective study on 251 Korean patients with PBC, age, Child–Pugh score, serum levels of TBIL, DBIL, ALB, Hb, and plasma creatinine, WBC and PLT counts, PT/INR, hemorrhage from esophageal or gastric varices, and hepatic encephalopathy were found to be associated with poor prognosis in patients with PBC [38]. However, these findings were not significant after adjusting for potential confounding factors. Nevertheless, in line with a previous report, lower WBC and platelet counts and prolonged international normalized ratio strongly predicted worse prognosis in PBC [39].

Corroborating several previous studies, we noted a definitive survival advantage in patients with PBC over those having PBC-SS and PBC-AIH. Cirrhosis in patients with AIH at any time during the disease course was found to be associated with a poor prognosis [40]. In addition, PBC patients with AIH features differed significantly from those without AIH features in terms of natural history, prognostic indicators, and response to therapy [36]. Further, a retrospective study of Chinese patients demonstrated that patients with PBC-SS had a lower survival compared with patients who had only PBC. Cirrhosis levels were significantly elevated in the PBC-SS group compared with the PBC group (*P*=0.003), which may be associated with a poor prognosis in the PBC-SS group. Taken together, our findings support the hypothesis that the prognosis of PBC may be dependent on the degree of severity of liver disease [38].

This study has several limitations. In this study, we enrolled only hospitalized PBC patients in a tertiary hospital that was specialized for liver diseases, which could lead to bias during patient enrollment. As this was a single-center study with a relatively small sample size, some serological parameters did not show statistical significance. Also due to the retrospective design of this study, potential uncontrolled biases which may confound the results were inevitable. In addition, further multicenter studies that are adequately powered with large sample sizes and long follow-up periods are required to detect significant associations between differences in outcomes. Moreover, the rarity of certain AIDs may have limited our ability to detect potential associations. A few findings of our study have not been observed in earlier studies, which may be attributed to differences in study design, sample size, characteristics of the study population, and/or the low prevalence of some autoimmune conditions.

In summary, we demonstrated the concomitant occurrence of other autoimmune entities with PBC and estimated their prevalence rates, in addition to those described earlier. In this study, SS was found to frequently coexist in patients with PBC. Patients with PBC-SS had significantly worse prognosis than patients with PBC alone. Despite being distinct autoimmune entities exhibiting unique clinical characteristics, autoimmune condition(s) coexisting with PBC may share similar pathogenic mechanisms. As PBC is a systemic disease and not organ-specific, screening for other autoimmune conditions in patients with PBC may prove beneficial. Further, identifying unique clinical and biochemical manifestations together with signs of specific organ involvement in concurrently occurring autoimmune disorders may aid in early diagnosis, treatment, and better prognosis in patients with PBC.

## Figures and Tables

**Table 1:** Clinical comorbidities in patients with primary biliary cholangitis (*n* = 505).

History of comorbidities	Number of PBC patients, *n* (%)
Hashimoto's thyroiditis	1 (0.2)
Graves's thyroiditis	3 (0.6)
Autoimmune hepatitis	36 (7.1)
Sjögren's syndrome	133 (26.3)
Hypothyroidism	4 (0.8)
Rheumatoid arthritis	7 (1.4)
SLE	2 (0.4)
AIH + SS	3 (0.6)
SS + SLE	1 (0.2)
SS + rheumatoid arthritis	1 (0.2)
AIH + Hashimoto's thyroiditis	1 (0.2)
SS + hypothyroidism	1 (0.2)
AIH + SS + RA	1 (0.2)

PBC: primary biliary cirrhosis. AIH: autoimmune hepatitis. SS: Sjögren's syndrome. RA: rheumatoid arthritis. SLE: systemic lupus erythematosus.

**Table 2:** Clinical features and laboratory results of patients with primary biliary cholangitis and those with primary biliary cholangitis complicated with Sjögren's syndrome.

Parameters	PBC group (*n* = 328)	PBC-SS group (*n* = 133)	*P* value
ALT (U/L)	56.60 (28.70–111.10)	54.20 (30.20–90.10)	0.419
AST (U/L)	70.30 (41.55–114.90)	74.20 (49.30–107.80)	0.714
TBIL (*μ*mol/L)	27.80 (14.90–75.30)	27.90 (18.30–64.30)	0.585
DBIL (*μ*mol/L)	15.00 (5.80–53.35)	13.50 (8.80–52.20)	0.512
TP (g/L)	70.60 (63.60–76.70)	70.20 (63.90–81.50)	0.313
ALB (g/L)	34.80 (29.60–38.80)	33.30 (29.70–36.80)	**0.008** ^*∗*^
GGT (U/L)	168.5 (76.9–358.8)	156.3 (118–305.6)	0.284
ALP (U/L)	189.30 (114.40–340.80) (114.40∼340.80)	245.8 (162.4–454.7)	0.558
WBC count (109/L)	4.63 (3.42–6.30)	3.22 (2.58–4.22)	**0.013** ^*∗*^
NE% (%)	59.06 (50.38–68.90)	58.8 (49.85–65.74)	0.682
Hb (g/L)	114.6 (98.60–128.00)	112.9 (79.8–121.8)	**0.003** ^*∗*^
PLT (109/L)	129.90 (77.30–196.20)	84.40 (76.00–124.00)	0.118
PT (s)	12.30 (11.10–14.10)	12.60 (11.50–13.80)	0.128
APTT (s)	34.30 (30.83–37.75)	36.25 (34.00–37.68)	0.321
UREA (mmol/L)	4.85 (3.82–6.58)	4.82 (3.76–6.23)	0.102
CREA (mmol/L)	57.00 (50.00–67.95)	56.00 (47.00–75.00)	0.374
IgM (g/L)	2.79 (1.58–4.25)	2.62 (1.47–5.10)	0.541
IgG (g/L)	15.40 (12.50–19.00)	17.70 (11.33–22.65)	0.111
AFP (ng/mL)	3.70 (2.58–5.50)	3.70 (2.00–5.40)	0.139
TC (mmol/L)	4.29 (3.39–5.49)	4.32 (3.29–5.13)	0.391
TG (mmol/L)	1.05 (0.66–1.58)	1.02 (0.66–1.54)	0.241
HDL-C (mmol/L	1.08 (0.60–1.51)	1.02 (0.46–1.27)	**0.015** ^*∗*^
LDL-C (mmol/L)	2.32 (1.69–3.04)	2.44 (1.74–2.83)	0.177
Ca^2+^ (mmol/L)	2.18 (2.06–2.27)	2.15 (2.08–2.25)	**0.004** ^*∗*^
PHOS (mmol/L)	1.08 (0.96–1.20)	1.11 (0.95–1.19)	0.116
ANA+	164/280 (58.6%)	76/125 (60.80%)	0.673
ACA+	60/319 (18.81%)	25/126 (19.84%)	0.803
SS-A/Ro-52kD+	95/287 (33.10%)	57/118 (48.31%)	**0.004** ^*∗*^
AMA+	218/312 (69.9%)	107/130 (82.3%)	**0.007** ^*∗*^
AMA-M2+	191/314 (60.80%)	101/129 (78.3%)	**<0.001** ^*∗*^
Fatigue	132/328 (40.24%)	44/133 (33.08%)	0.152
Pruritus	44/328 (40.24%)	12/133 (9.02%)	0.191
Cirrhosis	219/328 (66.77%)	107/133 (80.45%)	**0.003** ^*∗*^
Child–Pugh score	6.00 (5.00–8.00)	6.00 (6.00–8.00)	0.263
Child–Pugh score ≥7	141/328 (42.99%)	66/133 (49.62%)	0.194
Fibrosis-4	4.79 (2.37–8.93)	5.50 (2.99–9.29)	0.303
GLOBE score	1.83 ± 1.49	1.96 ± 1.52	0.507

PBC: primary biliary cholangitis; PBC-SS: primary biliary cholangitis complicated with Sjögren's syndrome; ALT: alanine aminotransferase; AST: aspartate aminotransferase; TBIL: total bilirubin; DBIL: direct bilirubin; TP: serum total protein; ALB: albumin; GGT: gamma-glutamyl transferase; ALP: alkaline phosphatase; WBC count: white blood cell count; NE%: neutrophil percentage; Hb: hemoglobin; PLT: platelet count; PT: prothrombin time; APTT: activated partial thromboplastin time; CREA: creatinine; IgM: immunoglobulin M; IgG: immunoglobulin G; AFP: alpha-1-fetoprotein; TC: Total cholesterol; TG: triglycerides; HDL-C: high-density lipoprotein cholesterol; LDL-C: low-density lipoprotein cholesterol; Ca^2+^: serum calcium; PHOS: serum phosphorus; ANA+: antinuclear antibody-positive; ACA+: anticentromere antibody-positive; SS-A/Ro-52kD: anti-SSA/Ro-52kD antibody-positive; AMA: antimitochondrial antibody-positive; AMA-M2+: antimitochondrial antibody-M2 mitochondrial subfraction-positive; ^*∗*^*P* value < 0.05 was considered significant.

**Table 3:** Clinical features and laboratory results of patients with primary biliary cholangitis and those with primary biliary cholangitis complicated with autoimmune hepatitis.

Parameters	PBC group (*n* = 328)	PBC-AIH group (*n* = 36)	*P* value
ALT (U/L)	56.60 (28.70–111.10)	71.2 (30.75–155.38)	0.208
AST (U/L)	70.30 (41.55–114.90)	90.55 (49.18–210.83)	0.079
TBIL (*μ*mol/L)	27.80 (14.90–75.30)	30.35 (17.73–155.56)	0.393
DBIL (*μ*mol/L)	15.00 (5.80–53.35)	16.10 (7.43–89.83)	0.368
TP (g/L)	70.60 (63.60–76.70)	72.05 (67.36–78.78)	0.174
ALB (g/L)	34.80 (29.60–38.80)	33.30 (27.35–37.48)	0.094
GGT (U/L)	168.5 (76.9–358.8)	121.70 (75.40–318.25)	0.416
ALP (U/L)	189.30 (114.40–340.80)	169.05 (101.33–289.08)	0.312
WBC count (109/L)	4.63 (3.42–6.30)	3.22 (2.58–4.22)	0.892
NE% (%)	59.06 (50.38–68.90)	58.8 (49.85–65.74)	0.338
Hb (g/L)	114.6 (98.60–128.00)	112.9 (79.8–121.8)	0.516
PLT (109/L)	129.90 (77.30–196.20)	122.30 (85.38–165.95)	0.886
PT (s)	12.30 (11.10–14.10)	12.80 (11.60–17.30)	**0.021** ^*∗*^
APTT (s)	34.30 (30.83–37.75)	35.70 (30.15–40.75)	0.398
UREA (mmol/L)	4.85 (3.82–6.58)	5.05 (3.76–6.11)	0.813
CREA (mmol/L)	57.00 (50.00–67.95)	58.80 (47.25–68.50)	0.833
IgM (g/L)	2.79 (1.58–4.25)	2.68 (1.43–4.35)	0.963
IgG (g/L)	15.40 (12.50–19.00)	16.45 (13.83–25.33)	**0.046** ^*∗*^
AFP (ng/mL)	3.70 (2.58–5.50)	4.25 (3.13–6.18)	0.052
TC (mmol/L)	4.29 (3.39–5.49)	4.44 (2.91–5.62)	0.65
TG (mmol/L)	1.05 (0.66–1.58)	1.14 (0.81–1.64)	0.264
HDL-C (mmol/L)	1.08 (0.60–1.51)	0.92 (0.40–1.28)	0.052
LDL-C (mmol/L)	2.32 (1.69–3.04)	2.36 (1.45–3.52)	0.822
Ca^2+^ (mmol/L)	2.18 (2.06–2.27)	2.15 (2.02–2.27)	0.327
PHOS (mmol/L)	1.08 (0.96–1.20)	1.05 (0.90–1.21)	0.268
ANA+	164/280 (58.6%)	26/33 (78.8%)	**0.025** ^*∗*^
ACA+	60/319 (18.81%)	5/35 (14.29%)	0.512
SS-A/Ro-52kD+	95/287 (33.10%)	13/34 (38.24%)	0.549
AMA+	218/312 (69.9%)	22/35 (62.9%)	0.394
AMA-M2+	191/314 (60.80%)	19/35 (54.3%)	0.453
Fatigue	132/328 (40.24%)	15/36 (41.67%)	0.869
Pruritus	44/328 (40.24%)	3/36 (8.33%)	0.388
Cirrhosis	219/328 (66.77%)	29/36 (80.56%)	0.092
Child–Pugh score	6.00 (5.00–8.00)	6.50 (5.00–9.00)	0.111
Child–Pugh score ≥7	141/328 (42.99%)	18/36 (50.00%)	0.421
Fibrosis-4	4.79 (2.37–8.93)	5.54 (3.02–9.50)	0.187
GLOBE score	1.83 ± 1.49	1.95 ± 1.47	0.400

PBC: primary biliary cholangitis; PBC-SS: primary biliary cholangitis complicated with Sjögren's syndrome; ALT: alanine aminotransferase; AST: aspartate aminotransferase; TBIL: total bilirubin; DBIL: direct bilirubin; TP: serum total protein; ALB: albumin; GGT: Gamma-glutamyl transferase; ALP: alkaline phosphatase; WBC count: white blood cell count; NE%: neutrophil percentage; Hb: hemoglobin; PLT: platelet count; PT: prothrombin time; APTT: activated partial thromboplastin time; CREA: creatinine; IgM: immunoglobulin M; IgG: immunoglobulin G; AFP: alpha-1-fetoprotein; TC: total cholesterol; TG: triglycerides; HDL-C: high-density lipoprotein cholesterol; LDL-C: low-density lipoprotein cholesterol; Ca^2+^: serum calcium; PHOS: serum phosphorus; ANA+: antinuclear antibody-positive; ACA+: anticentromere antibody-positive; SS-A/Ro-52kD: anti-SSA/Ro-52kD antibody-positive; AMA: antimitochondrial antibody-positive; AMA-M2+: antimitochondrial antibody-M2 mitochondrial subfraction-positive; ^*∗*^*P* value < 0.05 was considered significant.

**Table 4:** Univariate Cox proportional hazards regression analysis of prognostic factors for overall survival of patients with primary biliary cholangitis.

Characteristic/Parameters	RR	95% CI	*P* value
Gender	0.656	0.429–1.002	0.051
Age	1.028	1.012–1.044	**<0.001** ^*∗*^
Child–Pugh score	1.353	1.251–1.464	**<0.001** ^*∗*^
ALT	0.999	0.998–1.000	0.156
AST	1.001	1.000–1.001	0.234
TBIL	1.004	1.003–1.005	**<0.001** ^*∗*^
DBIL	1.005	1.004–1.007	**<0.001** ^*∗*^
ALB	0.912	0.885–0.939	**<0.001** ^*∗*^
GGT	1.000	0.999–1.000	0.648
ALP	1.000	0.999–1.001	0.480
WBC count	1.090	1.034–1.149	**0.001** ^*∗*^
Hb	0.986	0.979–0.994	**<0.001** ^*∗*^
PLT	0.994	0.992–0.997	**<0.001** ^*∗*^
PT/INR	3.706	2.639–5.204	**<0.001** ^*∗*^
CREA	1.011	1.007–1.014	**<0.001** ^*∗*^
IgM	0.954	0.885–1.029	0.222
AMA	1.339	0.861–2.083	0.196
Fatigue	0.708	0.488–1.026	0.068
Pruritus	1.204	0.725–1.999	0.473
Cholangitis	1.328	0.745–2.368	0.336
Esophagogastric varices bleeding	1.960	1.118–3.435	**0.019** ^*∗*^
Hepatic encephalopathy	2.569	1.680–3.927	**0.001** ^*∗*^

ALT: serum alanine aminotransferase; AST: serum aspartate aminotransferase; TBIL: total bilirubin; DBIL: direct bilirubin; ALB: serum albumin; GGT: serum gamma-glutamyl transferase; ALP: serum alkaline phosphatase; WBC count: white blood cell count; Hb: serum hemoglobin; PLT: platelet count; PT/INR: prothrombin time and International normalized ratio; CREA: plasma creatinine; IgM: serum immunoglobulin M; AMA: antimitochondrial antibody-positive; SE: standard error; RR: relative risk or risk ratio; ^*∗*^*P* value < 0.05 was considered significant.

**Table 5:** Multivariate Cox proportional hazards regression analysis of prognostic factors for overall survival of patients with primary biliary cholangitis.

Characteristic/parameters	RR	95% CI	*P* value
Age	1.010	0.992–1.028	0.269
Child–Pugh score	1.026	0.861–1.222	0.777
TBIL	0.995	0.980–1.011	0.541
DBIL	1.010	0.988–1.032	0.386
ALB	0.984	0.936–1.035	0.540
WBC count	1.072	1.016–1.130	**0.011** ^*∗*^
Hb	1.001	0.991–1.010	0.880
PLT	0.995	0.992–0.998	**0.003** ^*∗*^
PT/INR	1.799	1.010–3.206	**0.046** ^*∗*^
CREA	1.004	0.999–1.008	0.099
Esophagogastric varices bleeding	1.083	0.555–2.116	0.815
Hepatic encephalopathy	1.562	0.926–2.636	0.095

TBIL: total bilirubin; DBIL: direct bilirubin; ALB: serum albumin; WBC count: white blood cell count; Hb: serum hemoglobin; PLT: platelet count; PT/INR: prothrombin time and international normalized ratio; CREA: plasma creatinine; SE: standard error; RR: relative risk or risk ratio; ^*∗*^*P* value < 0.05 was considered significant.

## Data Availability

The data used to support the findings of this study are included within the article.
